# Ecological Disasters and Mental Health: Causes, Consequences, and Interventions

**DOI:** 10.3389/fpsyt.2020.00001

**Published:** 2020-02-11

**Authors:** Joshua C. Morganstein, Robert J. Ursano

**Affiliations:** Department of Psychiatry, School of Medicine, Uniformed Services University, Bethesda, MD, United States

**Keywords:** disaster, mental health, trauma, vulnerable populations, early interventions

## Abstract

Ecological disasters highlight the importance of understanding natural disasters as they relate to a changing global climate. Such disasters often have a predictable pattern of evolving over time and anticipated psychological and behavioral problems and community disruptions. Various factors enhance transmission of these adverse effects beyond the geographic location of the ecological disaster, with certain populations being particularly vulnerable to these effects. Understanding the range and pattern of these effects can aid in optimizing interventions. The use of evidence-informed interventions can reduce distress, enhance well-being, and improve functioning for affected individuals and communities. Effective preparedness involves an understanding of these factors, incorporation of them at all stages of disaster management, and continuous education and training for disaster planners and responders.

## Disasters and Public Health

**Figure 1:**
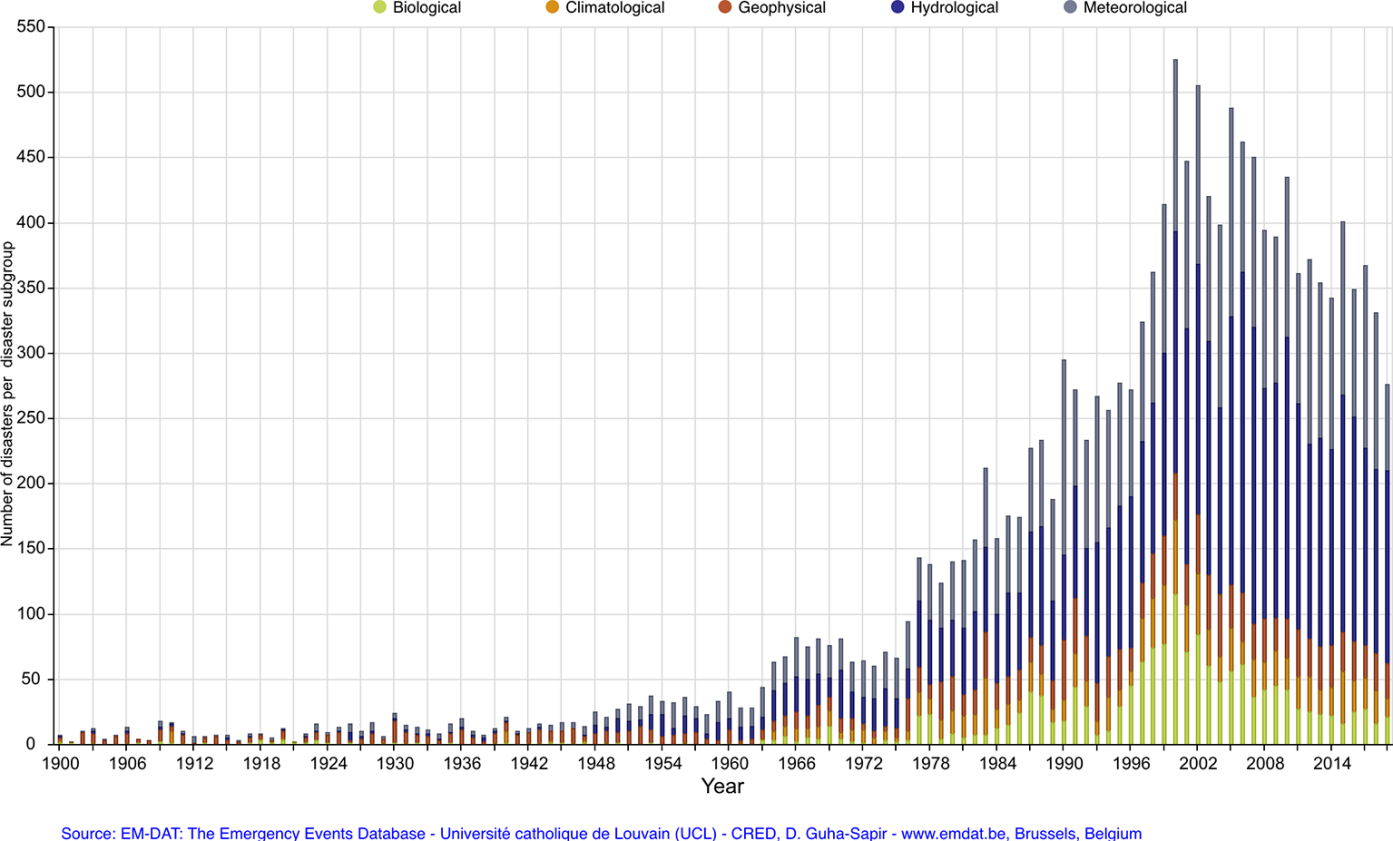
Frequency of climate-related disasters by type and year.

Disasters are severely impacting events that overwhelm the coping resources of a local community. Ecological disasters may be abrupt and extreme weather events that unfold over minutes or hours (tsunami, earthquake, hurricane) or slow-moving events that span days, weeks, or months (floods, droughts, wildfires). Ecological disasters are occurring with increased frequency and severity (see **Figure 1**), believed to be in part due to a changing global climate, which has been called one of the most significant threats to global health in the 21^st^ century (1). These disasters result in disruption through damage to property, physical injury and death, psychological distress, displacement of individuals and families, and prolonged disruption to a broad range of services upon which communities rely.

The increased frequency of ecological disasters furthers the need for enhanced planning efforts to mitigate the adverse psychological and behavioral effects of these events. While there remains debate about the degree to which future climate-related disasters may be mitigated through human intervention, it is clear that the increase in these events will necessitate more resourcing by policy makers, rigorous planning by disaster managers, education of personnel involved in response and recovery, and research to more effective articulate optimal timing and sequence of interventions. All-hazards planning addresses the full spectrum of threats, including all types of disasters, and is the current framework for global disaster management with mental health being recognized as a critical aspect of healthcare that should be incorporated into all phases of disaster planning (2).

Communities (neighborhoods, schools, workplaces, healthcare facilities) have unique needs that benefit from thoughtful planning and preparedness. Effective preparedness improves response and recovery following a disaster and may reduce overall resource requirements. Disasters strike at the fault lines of communities by laying bare and exacerbating sociocultural divisions within the unique contextual factors of a given community. Various factors impact the community experience of a disaster (see **Table 1**). These and other factors create a disaster ecology in which various forces of harm impact individuals, communities, and societies (3). For example, cultural and contextual factors were critical to community partnership and effective response in: 1) Haiti earthquake in 2010 required knowledge about and incorporation of voodoo as a religious ritual integral to how citizens conceptualized and responded to healthcare interventions (4), 2) West Africa Ebola virus outbreak in 2000 necessitated awareness and collaboration with local communities to incorporate the importance of faith-based burial rituals (5), 3) Flint, Michigan lead water crisis in 2015 was experienced by community members as further evidence of systemic racial inequities that further eroded public trust (6). Each of these events, as with all disasters, necessitated an understanding of sociocultural and contextual factors within the communities to optimize response and recovery efforts.

**Table 1:** Factors impact community experience of disaster.

Prior exposure to disasters
Pre-existing socioeconomic resource disparities
Religious and cultural beliefs about the meaning of the disaster
Trust in government institutions (elected officials, law enforcement, aid organizations)
Prior experiences with national and/or international government intervention
Presence of litigation

Disasters impact large and diverse populations. How the psychological response is managed is, perhaps, the most critical factor in a community's ability to recover. Effective interventions are rapid, coordinated, and sustained. Leadership is critical, particularly knowledge of community resilience and vulnerability as well as how community members respond to the event. Coordinated approaches across public health, medical, and emergency response is optimal to address mental health needs of the disaster affected population. All hazards planning focuses on the preparedness measures that address the full range of threats for communities, including both natural and human-generated disaster events with consideration of various impacts on a range of effected populations. The Haddon Matrix is an often-used framework for risk analysis and mitigation that considers the host, agent/vector, physical and social environments across the pre-event, event, post-event time periods (see **Table 2**). The use of an established framework to consider the range of factors impacting various phases of disaster ensures preparedness activities are structured and comprehensive; advanced planning reduces the chance that disaster managers make errors or miss important factors during the high stress environment of crisis response following disaster impact. Effective planning reduces distress for affected personnel and community members and optimizes access to needed mental health care following the event.

**Table 2:** Haddon matrix applied to an earthquake.

PHASE	INFLUENCING FACTORS		
	Agent: (Earthquake)	Vector: (Natural Disaster)	Population (Community)
Pre-Event	- Response training - Seismology monitoring - Public education	- Earthquake monitoring systems	Preparedness Behaviors: - Risk assessment - Information/Plan - Surge capacity planning
Event	- Leadership - Early warning systems - Risk communication	- Emergency medical training - Early detection of event	Disaster Behaviors: - Active coping - Evacuation - Hospital surge capacity
Post-Event	- Emergency response - Ongoing communication	- Response and recovery systems and infrastructure - Enhanced warnings	Response/Recovery Behaviors: - Community leadership - Psychological First Aid - Psychotherapy - Medications

“Tipping points” may occur following disasters. The term “tipping point”, frequently used in the field of climate science and popularized by Malcolm Gladwell, describes a phenomenon whereby a small change in the balance of a system results in a relatively large downstream effect (7). A variety of factors may provoke tipping points in community response to a disaster (see **Table 3**). The result is a significant increase in community distress, which may be associated with reduced adherence to recommended health behaviors. This may lead to increased strain on public health systems and suboptimal utilization of health resources, ultimately worsening community well-being and prolonging recovery. While certain events may serve as tipping points, their occurrence is the result of a complex interplay of social, cultural, and contextual factors. As a result, tipping points cannot always be avoided. However, clear and consistent communication, equitable distribution of resources, and active community engagement may reduce the likelihood of tipping points and their associated adverse psychological and behavioral responses. Community leaders should remain vigilant for tipping points, take steps to reduce their likelihood of occurrence, as well as recognize and mitigate the impact when they occur.

**Table 3:** Tipping point events following disasters.

Sudden belief that resources are inadequate, ineffective, or unfairly distributed
Development of conspiracy theories and rumors
A sudden, intentional and unexpected event (ie, terrorism)
Loss of faith in social institutions and community leaders
Restricting of civil liberties (particularly if perceived to be done evenly)
Death of children or other populations considered particularly vulnerable

Psychological and behavioral health consequences represent the overwhelming majority of not only human suffering, but overall healthcare expenditure, following ecological disasters. Cost modeling estimates following Hurricane Katrina, which occurred in the United States in 2005, suggested that screening and evidence-based treatment for common mental disorders in the affected population would nearly equal the cost of restoring the failed levy system around New Orleans (which was the source of catastrophic flooding leading to property damage as well as the majority of injuries and deaths) (8, 9). While this article will focus primarily on the human impacts of ecological disasters, it is important to understand that effectively articulating the economic benefits of preparedness can serve as a crucial data point to further assist government officials in financial decision-making and resourcing of disaster preparedness.

## Consequences

Most people will do well following a disaster, recovering promptly to previous levels of function. This does not mean they are not affected, but they remain effective in their work and families and adapting to the situational needs. Some may experience an increased sense of efficacy and the belief in their ability to manage future challenges, often termed post-traumatic growth. However, some will also experience adverse mental health effects of ecological disasters. The psychological effects of disasters begin immediately following the event and may persist for extended periods of time, extend beyond the geographic region directly impacted by the event, and are experienced within the broader culture and context of a community.

### Psychological and Behavioral Effects

**Figure 2:**
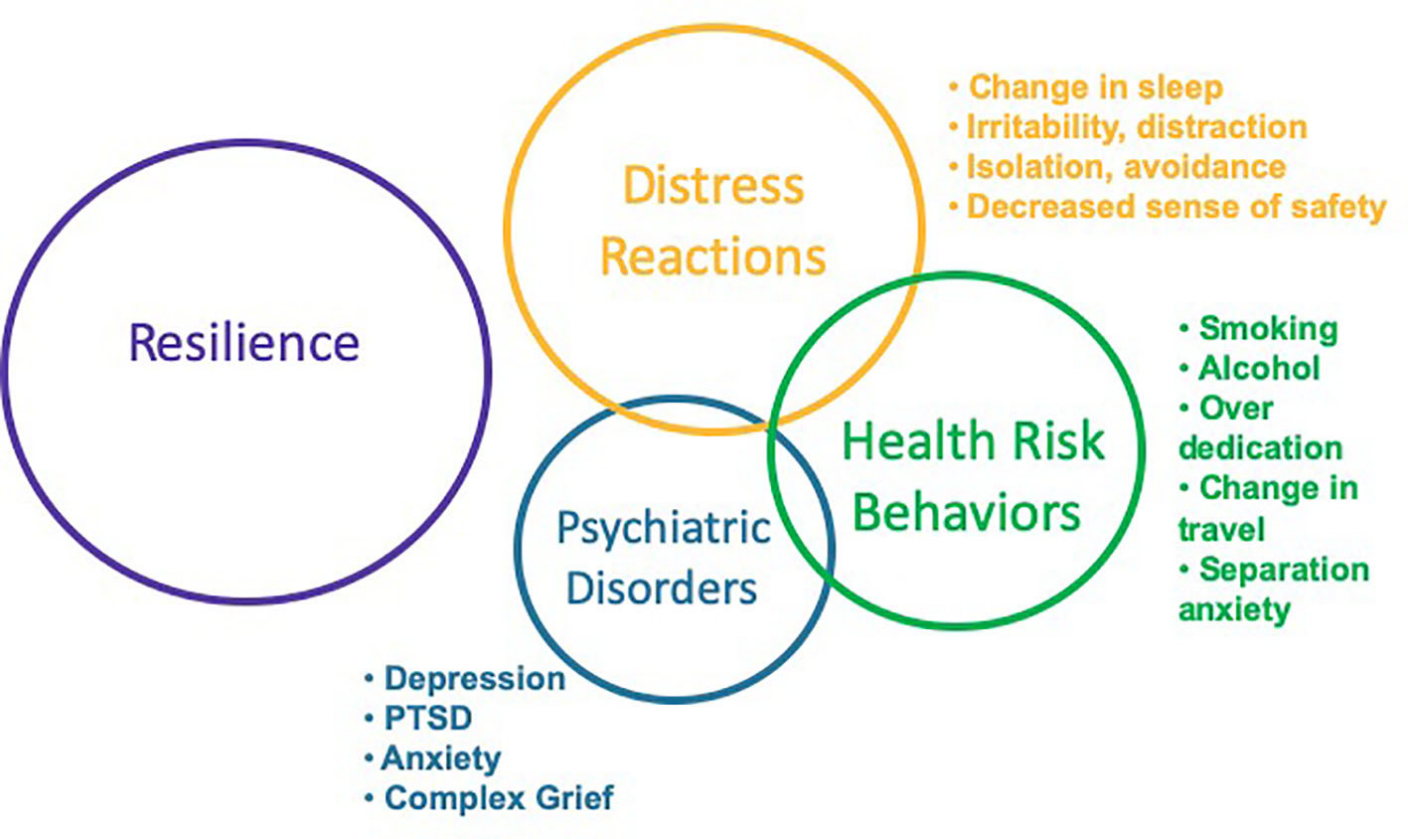
Psychological and behavioral responses to disasters.

Considerations of adverse psychological and behavioral effects often focus on psychological disorders, such as Posttraumatic Stress Disorder, Depression, and Anxiety. These disorders do occur following disasters and result in considerable morbidity and mortality, warranting prompt assessment and evidence-based interventions. In addition to disorders, earlier and more common responses include distress reactions and health risk behaviors (see **Figure 2**) (10). In healthcare settings, concerns such as insomnia, anxiety, and altered substance use patterns are most commonly identified in primary care and emergency settings. It is important for healthcare systems to ensure adequate education and resourcing of primary care and emergency personnel to manage the predictable responses following ecological disasters. Understanding the broad range of adverse effects will enhance planning and preparedness, as well as response and recovery efforts, by disaster managers, healthcare personnel, and community members. It is important that education and training for healthcare personnel and disaster managers continue to evolve beyond contemporary approaches of diagnosing and treating illness. Instead, there should be a transition to early screening and delivery of public health interventions that are evidence-based, cost-effective, readily accessible, and community-focused with the goal of reducing distress, enhancing well-being and functioning, reducing the rate of progression to psychological disorders and, ultimately, improving the overall trajectory of community recovery following disasters.

Distress reactions are early and common manifestations following traumatic events, representing the bulk of early public mental health burden following ecological disasters. Insomnia is highly prevalent and increases risk for other psychosocial difficulties (11, 12). Following the Wenchuan earthquake in 2008, 38.3% of adolescents reported rates of sleep disturbances at 12 months after the event with no significant reduction at 24 months post-event; sleep disturbance was associated with increased rates of depression and PTSD (13). Anger is common following disasters and associated with increased likelihood of negative mental health outcomes (14). Demoralization, loss of faith, distractibility, and decreased perceptions of safety may also occur. Distress reactions are often the result of a complex interplay between various factors in the post-disaster environment. For example, following a flood, hurricane, or tsunami, individuals may be forced to flee their homes, and many will need to reside in shelters, makeshift lodging, or camps. These facilities are often cramped, noisy, and lack adequate security measures. This environment reduces feelings of safety, making it more difficult to sleep, resulting in chronic fear-induced insomnia. Insomnia, in turn, reduces cognitive ability to manage negative emotions like anger, increases distractibility, and diminishes capacity for the critical skill of problem-solving. Public health interventions that address common aspects of the disaster environment that exacerbate distress reactions will increase the efficacy of affected community members and more quickly restore functioning and productivity.

Health risk behaviors are maladaptive coping strategies to manage distressing emotions and include increased use of alcohol use (15) and tobacco (16). Increased use of alcohol increases risk for motor vehicle accidents and family violence. In addition to an increased use of substances to cope with distressing emotions, some people may even begin using these substances for the first time. In a study of 37,867 individuals who were non-drinkers prior to the Japan triple disaster in 2011, 9.6% reported starting drinking in 2012; among those who started drinking, 53.8% continued drinking in 2013 (17). Individuals may also isolate themselves, reducing access to available healthcare and social support resources (18). Over-dedication can occur when individuals spend excess time at work or on recovery efforts as a means of distracting from other important social and occupational challenges that warrant more urgent attention. Primary care/general medical physicians and emergency department personnel can provide screening and educational interventions to identify and mitigate high risk health behaviors. Public health messaging from community leaders and the media can also provide education information, including high risk health behaviors to avoid, alternative coping mechanisms, and where to get help if needed.

Psychological disorders may also develop following ecological disasters, produce significant morbidity and mortality, and require healthcare interventions. The most studied is Posttraumatic Stress Disorder (19, 20), along with depression (21, 22) and anxiety (22, 23). A study of residents in Mexico 2 months after the September 2017 earthquake revealed 36.4% indicating symptoms consistent with PTSD, with increased risk found in women, those who had home damage, and indigenous people (24). Previous psychological disorder increases risk of recurrence following a disaster. Screening of the affected population and, when indicated, prompt assessment and initiation of evidence-based interventions represent clinical best practices.

The recovery period following ecological disasters is often stressful and prolonged. As health, financial, occupational, and family stressors mount, coping resources may be exhausted. Suicidal thoughts and behavior increase after disasters and result from a milieu of pre- and post-disaster factors that overwhelm the ability to cope (25–27). Of note, some research has observed suicidal thoughts and behavior to diminish moderately from baseline in the early weeks and months following a community disaster (consistent with the “Honeymoon” phase of community recovery; see below), but then increase from baseline during the ensuing months and years. Interpersonal violence has been shown to increase, with women being most effected (28, 29). In addition, population displacement and migrations, breakdown of community infrastructure, food scarcity, loss of employment, and poor sense of social connectedness have negative consequences for psychological and behavioral health (30, 31).

#### Children and Adolescents

The psychological and behavior response of children and adolescents may include those observed in adults as well as other reactions based on developmental stage and other factors. However, responses may look different and can be easily overlooked or misinterpreted as acting out behavior when observed by stressed and distracted parents, educators, and school administrators (32). Behaviors more unique to children and adolescents that may indicate adverse response following ecological disasters include regression, diminished academic performance, aggression, and self-blame (33). Separation from primary attachment figures, parental distraction and family strife, and disruption in schedules and routines are factors that increase vulnerability for children and adolescents. Education and support resources for parents, teachers, and other school personnel can help more effectively identify distress reactions in youth, allowing for more timely and effective interventions.

#### Grief

Grief is a near universal reaction following ecological disasters and occur in response to profound loss. Losses include not only of loved family or friends but also of one's home and cherished mementos, such as photographs and items handed down from previous generations. Those who are displaced can also lose their community and its support, comfort with familiar surroundings, pets, and their usual life routines. Individuals and families in evacuation centers and shelters can also lose their sense of safety and security, home comforts, and the restoration that comes with routine sleep. Traumatic grief increases the likelihood of adverse mental health outcomes. Bereaved Norwegian family members who lost a loved during the 2004 Asian tsunami found that, 6 years after the event, 36% had a psychiatric disorder and the presence of prolonged grief disorder independently increased the risk for functional impairment (34).

#### Exposure and Contamination

Ecological disasters can also result in infrastructure damage that can create risk for exposure and contamination by chemical, biological, radiological, or nuclear (CBRN) material. These require unique public health preparedness measures (35). Overflowing waste treatment plants, damage to nuclear or biological facilities, and even human corpses inadvertently exhumed by extreme weather events can result in psychological and behavioral responses within effected communities (36). Following the earthquake and tsunami on the island of Honshu, Japan in 2011, damaged reactors in Fukushima exposed the community and the surrounding soil and water to nuclear material. The resulting fear and uncertainty about nuclear contamination led to ostracizing and hostility toward displaced individuals most severely affected (37). CBRN materials are perceived as dangerous, mysterious, undetectable, and novel to citizens, disaster managers, and even many healthcare providers. Uncertainty about exposure, fears of prophylactic medication and treatment shortages, as well as concerns about isolation and quarantine fuel distress and can increase risk for community fear (38).

CBRN events result in high levels of anxiety-related somatic symptoms, often referred to as Medically Idiopathic/Unexplained Physical Symptoms (MIPS/MUPS). Risk of CBRN exposure highlights the importance of the perception of risk, which is higher than actual risk. As a result, public health messaging is critical to educate the community regarding true risks, steps being taken to mitigate risks, as well as when and where to get help (39). Healthcare facilities should be prepared to manage and triage to appropriate care citizens with high levels of somatic concerns related to fear of exposure. Mental health personnel trained in the effects of mass trauma and evidence-based interventions, embedded in emergency and primary care/general medical care settings, can provide support and initiate early interventions to reduce distress.

During infectious disease outbreaks, absenteeism among healthcare personnel due to their own concerns or needs to care for family can further reduce needed resources during times of increased demand for care. Healthcare personnel distress is fueled by fears of becoming ill, being ostracized by family and friends, and concerns about adequacy of protective equipment, increasing rates of absenteeism (40). At a U.S. medical center in 2006, only 50% of surveyed respondents indicated “yes” when asked if they would report to work during an Avian flu outbreak (41). It is important for healthcare facilities to reduce barriers for healthcare personnel reporting to work and ensure adequate resourcing during times of increased care demand. An extensive qualitative review of healthcare organizations conducted by the U.S. Department of Energy revealed that measures most likely to reduce fear-based absenteeism included effective early and ongoing communication by hospital leadership, ensuring adequacy of personal protective equipment, and maintaining adequate staffing to avoid overburdening healthcare personnel (42).

#### The Role of Media

Media can be a help for disaster affected communities, providing information on risks, recommended health behaviors, as well as knowing when to get help and how to access resources. Alternatively, media can be a source of rumors and added fear. While most studies of media exposure have examined impacts following acts of terrorism and mass violence, a number have reviewed the impact following ecological disasters. This research has consistently found that increased exposure to media after disasters is associated with adverse outcomes (43, 44). In this way, media serves as a vector of distress transmission. High media exposure may also be a way of attempting to control or alleviate anxiety in those already experiencing higher levels of distress.

Elected officials, community leaders, responders, and healthcare personnel may all engage with media during disaster response and recovery. The media will understandably expect information from those involved in disaster management. It is helpful to work collaboratively to ensure accurate information is conveyed and important public health information disseminated. Abruptly displaying graphic content can enhance distress for viewers. Encourage the media to provide warnings before showing graphic material and indicate the date of material being shown; the latter helps people to know if the event has already taken place and does not represent a new event that might generate unnecessary fear.

Social media is increasingly used following disasters and patterns have been observed in both the patterns of social connectivity as well as focus of information sought by users during different phases of disaster providing important information to guide disaster communication and messaging (45). In addition to use of social media for information following disasters, an increasing array of online and mobile resources are available to enhance disaster preparedness, response and recovery for responders and community members impacted by disasters (see **Table 4**). They can be used to provide critical guidance on sheltering in place, evacuations, where and when to access available resources, as well as preparedness and response guidance for specific disasters, such as hurricanes or hazardous material spills. Mobile apps can be used to crowd source data about the direct impact of disaster events down to the individual level, such as home damage and physical injury, as well as information about services that remain operational accessible for those in need. Extreme weather events often adversely impact access to electricity as well as internet and mobile device connectivity, so reliance solely on these devices for needed resources should be avoided.

**Table 4:** Mobile apps for disasters.

FEMA	Access to weather services and disaster preparedness and response tips, local shelters, location of FEMA disaster recovery centers; also allows submission of photos of disaster damage
SAMHSA Disaster Behavioral Health	Information and resources on disaster behavioral health issues relevant to preparedness, response and recovery. Info sheets that can be downloaded directly to the device allowing access during cellular signal disruption
WISER	First responder Hazmat incident resource; helps identify substances, containment and suppression advice, medical treatment information
Nextdoor	Allows user to indicate they are in distress and any local users will be provided their location to come provide assistance
GasBuddy	Provides information on closest gas stations that are operational and able to provide gas
Zello	Walkie-talkie app that allows faster communication and sending of photos and voice messages
Life360	Allows families to locate and monitor one another's location; “panic” function sends message and email to all family members at once

Research on the impacts of media on psychological and behavioral responses following disaster has examined television to a much greater extent than social media. Future research will elucidate how the increasingly dominant news platform of social media impacts communities after disasters. A broad range of opportunities exists to use social media to gather critical post-disaster information through crowdsourcing as well as dissemination of important response and recovery content through mobile devices. Challenges to understanding the role of social media as well as capitalizing on potential benefits of this medium of communication will include the increasing polarization of sociopolitical and cultural views, mistrust of institutions including media, the impact of data breaches, and the ways in which the burgeoning field of artificial intelligence increasingly allows for dissemination of disinformation.

## Community Phases

**Figure 3:**
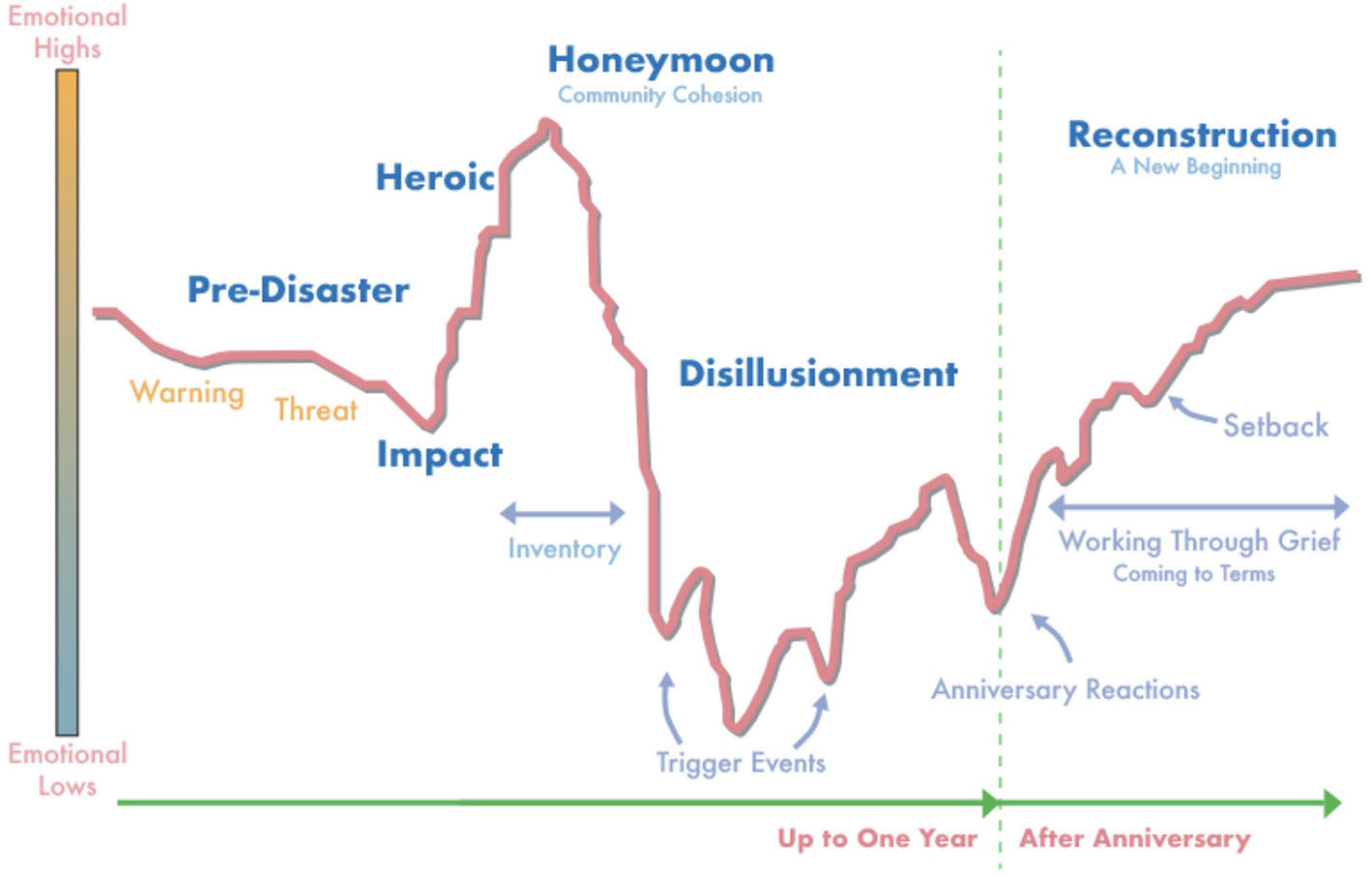
Community phases of recovery following disaster (47).

Following an ecological disaster, particularly those that involve a single acute event (such as tsunami, earthquake, or hurricane), affected communities often progress through six phases of psychosocial recovery (see **Figure 3**). These phases have been articulated less by empirical literature and more so by considerable field experience of disaster mental health experts. Several of the phases have particular relevance for understanding community response and consideration of disaster planning and resource allocation.

The Honeymoon phase coincides with increased availability of government, volunteer, as well as international assistance. Community bonding occurs through a shared catastrophic experience as well as giving and receiving of assistance. Survivors are more hopeful and optimistic the help they receive will restore them to wholeness. Disaster mental health workers are more accepted by community members and can develop a foundation on which to provide assistance in difficult phases ahead. As previously noted, rates of suicide attempts may decrease during this time period, presumably due to increased support resources as well as innate characteristics of affected individuals (46).

The Disillusionment phase is characterized by disappointment as disaster assistance agencies and volunteer groups pull out of the community and hopes for restoration to pre-disaster emotional and physical wellness go unmet. The sense of community is weakened as people focus more on unmet needs. Resentment may surface as survivors receive unequal monetary compensation for what they perceive as similar damage. Less impacted neighboring communities have returned to life as usual, which can discourage and alienate those more severely impacted. Survivors may become physically exhausted due to growing multiple demands, including financial pressures, relocation or living in a damaged home, family discord, bureaucratic hassles, and a lack of free time for recreation or self-care. Health problems and exacerbation of pre-existing conditions emerge as a result of ongoing stress and fatigue. The disaster “Anniversary” experience occurs during this phase and serves as a critical opportunity for leaders to support the psychological well-being of disaster victims through memorializing, making meaning, and “building back better”. Failure to address a disaster anniversary experience can further demoralize survivors, exacerbate underlying psychological distress, and worsen the trajectory of community recovery.

The Reconstruction phase often lasts for years. Survivors attempt to rebuild their lives as well as social and occupational identities by rebuilding homes, returning to old jobs or finding new work, and resuming or establishing new social support systems. Some are able to accept new circumstances, including losses and changes that have occurred. Individuals may find meaning and ultimately emerge with an increased sense of personal strength and belief in their ability to manage future adversity. Others may instead focus more on resentment, anger and scapegoating, choosing to reshape their identity as a victim. Individuals progress through these phases at different times. Consequently, disaster planners and victim service providers should recognize that persons manifest various emotional symptoms over different timelines in response to the same event. Moreover, depending on the severity of the experience, the resources available during and after the event, and individual's coping skills, varying numbers of individuals develop persistent symptoms requiring prolonged treatment. Anger may be directed at caregivers and community leaders if these factors are not sufficiently accounted for in medical and psychological response plans.

It should be noted that this model of community recovery was developed within the context of disasters occurring in the United States and may have greatest applicability to developed countries. In developing nations, a government's resources and the willingness and ability of leaders to aid citizens, degree of trust citizens have in the government, and the presence of national and international non-governmental organizations impact the extent to which a community progresses through these phases of recovery. For instance, if a government lacks the financial means to provide resources for severely impacted citizens, a Honeymoon phase may not occur because a sense of despair is never alleviated by the influx of meaningful assistance. In this case, **Figure 3** might be altered to show an emotional high that steadily diminishes following the Heroic phase and never improves. Slowly evolving disasters may cause the Impact phase to be drawn out, delaying the community cohesion and sense of togetherness that comes from the Honeymoon phase. In addition, when citizens perceive the threat of contamination with CBRN agents, the Honeymoon phase frequently does not occur as community members limit contact with one another due to fear of exposure. When community leaders disregard the predictable Anniversary reaction, community distress may increase and well-being diminish, delaying or preventing progression to the Reconstruction phase where citizens make meaning and move on with their lives. Disaster planners and community leaders can enhance the effectiveness of response and recovery efforts by anticipating and planning for factors that impact community phases of recovery.

## Vulnerability to Disasters

Increased vulnerability to psychological and behavioral effects is the result of various factors, including pre-event characteristics, event impact, and recovery variables (see **Table 5**). Various populations are at increased risk for adverse mental health effects of ecological disasters and warrant special consideration in disaster preparedness, response, and recovery.

**Table 5:** Factors increasing vulnerability to mental health effects of disasters.

Pre-Event Characteristics	Event Impact	Recovery Variables
Socioeconomic status	Duration & severity of exposure	Relocation
Age	Physical injury	Job loss
Gender	Home loss	Social support loss
Social support	Displacement	Victim litigation
Reliance on care systems	Bereavement	Financial stress

Lower socioeconomic status is often associated with worse outcomes following disasters (48). Those with less financial resources often reside in locations that are more prone to and less resistant against the effects of ecological disasters (49). Hurricane Katrina in 2005 and the Indonesia tsunami in 2004 exemplified the increased vulnerability to psychological and behavioral harm, destruction, and death conferred on those of lower socioeconomic status. Diminished financial resources are associated with increased rate of homelessness, reduced preparedness behaviors, diminished ability to evacuate or avoid the impact of disasters, limited access to needed health care resources following a disaster, as well as increased psychological distress and posttraumatic stress symptoms (50). Those experiencing homelessness have additional challenges, including lack of access to disaster preparedness information, decreased means of communication, absence of a physical dwelling structure for protection, and high rates of chronic medical and mental health conditions (51).

Individuals with pre-existing mental health conditions, particularly those with active and severe symptoms, have needs following large-scale community disasters (52). Like others, most with mental health conditions will rise to the occasion and participate in disaster response efforts. However, people with mental health conditions may be less prepared for disasters than others (53). Failure to continue or resume care has been observed with much greater frequency following mass trauma events, such as ecological disasters (54). In addition, those who are reliant on systems of care for medical monitoring and ongoing interventions are at risk when there is damage to infrastructure and delays in medication supply chains. Individuals taking psychotropic medications may experience diminished heat regulation and impaired fluid homeostasis during extremes of temperature, resulting in adverse medical events (55). Higher rates of poverty, sub-standard housing or homelessness, diminished community infrastructure, and co-occurrence of substance use disorders all amplify risk for individuals with mental illness.

The increased vulnerability of children is well-established. Research has focused primarily on PTSD, though anxiety, depression, behavioral disruption, and substance use have also been observed. Parental loss or psychopathology following a disaster event are important predictors of child well-being (56). In addition, the quality and style of the parenting relationship are important aspects of child vulnerability (57). Social support and life stressors may impact well-being in children to a greater degree than extent of disaster exposure (58).

Literature on the vulnerability of elderly to disasters is inconsistent, with some studies revealing increased rates of adverse mental health outcomes (59). In some instances, age may serve as a protective factor, with those of advanced years having significantly greater life experience navigating adversity and enhanced overall stress resilience. Overall, research suggests that conditions frequently associated with advanced age (cognitive difficulties, vision impairment, mobility limitations, and chronic health conditions resulting in dependence on medical equipment and systems of care), rather than age per se, create the majority of risk (60).

First responders and public health emergency workers are highly exposed to disaster related traumatic events and many have increased mental health symptoms following disasters (61, 62). These individuals often have considerable burden in the response and recovery phase of disaster events. They frequently work long hours, may be exposed to severe injuries and mass death, are often under consider pressure to perform, and may themselves be disaster victims who are unable to adequately attend to the needs of their family or provide for self-care. Psychological “identification” with human remains (“that could have been me” or “that could have been my child”) increases risk for adverse psychological effects (63). A study of first responders involved in Hurricane Katrina, including police, fire, emergency medical, and city workers, revealed that, at 6 to 9 months after the disaster, 40% reported increased use of alcohol and 25% reported significant levels of depression; the latter rate persisted without any evidence of decreasing at 18 months post-disaster (64).

In some studies, women have been found to be at increased risk for adverse psychological outcomes following disasters (65). There are several factors which may contribute to this finding. Women are often increasingly burdened with managing the distressing social dynamics that occur in a family impacted by disaster. Intimate partner violence increases in both frequency and severity following disasters, with victims predominantly being female (66). In communities effected by civil unrest and war, women more commonly than men experience poly-victimization as a result of theft, physical assault and sexual harm. Pregnancy and post-partum state also increase risk (67, 68).

## Interventions for Community Recovery

Early, effective, sustained interventions following disasters aid recovery and optimize community functioning. Important considerations include prevention measures, barriers to care, and the role of leadership. Assessment should examine a broad range of behavioral and psychological reactions to traumatic events as well as level of impairment. Evidence-based interventions focus on reducing distress, enhancing well-being and optimizing social and occupational function.

### Assessment

Following ecological disasters, many individuals will manage distress without intervention or will rely on community support from neighbors, friends, and family. Those who experience significant, prolonged or impairing distress need timely assessment by personnel trained to understand the unique effects and comorbidity associated with disaster-related trauma. Community education about normal reactions and when to get help can assist in triaging those who require medical interventions. This process is enhanced by collaboration with media, local social and religious organizations, as well as training for first responders and healthcare personnel. Most people who seek healthcare will present to emergency and primary care settings. In some communities, distress will be seen as a spiritual or religious concern with faith leaders having a prominent role in managing community well-being. For outside organizations (such as NGOs and international aid providers) understanding the culture and values of a local community facilitates partnership and engagement in health-promoting behaviors following a disaster. Personnel working with community members should be trained to assess for the full range of psychological and behavioral responses that may occur and refer those needing interventions for appropriate care. When assessment personnel are inadequately resourced, they may become overburdened and unable to provide the time, energy, and continuity of support to assist people in need.

Assessment should consider not only the presenting concern or specific traumatic event, but the individual's entire “network of stressors” (see **Table 6**). Any additional stressor exacerbates the primary distress of the event, adding to an individual's overall burden of distress. Individuals will predominantly manifest distress reactions, health risk behaviors, and less frequently psychiatric disorders (see **Figure 3**). A focus exclusively on making a psychiatric diagnosis will often overlook a range of psychological and behavioral responses contributing to significant distress and functional impairment. Health care assessment can include clinical interview with a healthcare provider as well as the use of standardized scales for trauma and comorbid illnesses such as the PTSD Check List (PCL-C), Patient Health Questionnaire-9 (PHQ-9) for depression, Alcohol Use Disorder Identification Test (AUDIT-C). Intensity and duration of exposure to disaster-related trauma should be ascertained, as this can help determine the likelihood an individual will develop distress reactions.

**Table 6:** Network of stressors to be considered during evaluation.

Living instability (displacement, stress of evacuation center)
Medical and mental health conditions (chronic pain, depression, anxiety, grief)
Substance use and misuse (alcohol, prescription medication)
Occupational challenges (loss of job, inadequate resources, overworked)
Family challenges (geographic separation, intimate partner violence)
Other social difficulties (legal, financial, neighborhood)

Evaluation for comorbidities associated with traumatic stress may reveal additional symptoms or disorders, which complicate treatment planning. Symptoms of depression, anxiety, and substance use should be elucidated and considered in the process of developing interventions. Somatic complaints may also be common, such as headache, indigestion, dizziness, or palpitations, among other symptoms. These are easily overlooked, particularly by those performing evaluations who may lack medical training. When physical symptoms predominate, increased social and occupational impairment may occur. Ensuring community members are aware of helping resources and encouraging them to utilize these before impairment worsens can be a helpful intervention. Care should be taken to reduce the degree to which the assessment of disability serves as a stigmatizing event and barrier to effective care for the individual.

### Interventions

Early interventions address distress reactions and health risk behaviors in which the primary goals are to reduce adverse effects, preserve functioning and decrease progression to psychiatric disease. When psychiatric disorders occur, evidence-based psychotherapy and pharmacotherapy may help reduce symptoms and functional impairment. Complementary and alternative interventions have an increasing body of knowledge supporting their use in the treatment of traumatic stress. A range of behavioral self-help interventions may be used throughout. Many individuals will prefer social and community support over formal intervention. A comprehensive treatment plan involves the use of interventions which address the unique circumstances of the trauma in the context of the individual's preferences (see **Table 7**).

**Table 7:** Interventions for trauma-related symptoms following disasters.

Psychological First Aid (safety, calming, efficacy, connectedness, hope/optimism)
Self-help interventions
Peer support
Trauma-focused psychotherapies (CPT, PE, SIT, EMDR)
Pharmacotherapy (focus on regulating sleep and promoting calming; short-term use)
Complementary and alternative interventions (yoga, meditation, mindfulness)
Behavioral interventions (diaphragmatic breathing, muscle relaxation, imagery)

Those managing disasters should understand the resources available to their community following a disaster. This will be heavily influenced by the type and extent of disaster impact. Disasters that destroy infrastructure and displace large numbers of citizens often severely constrain local resources, increasing reliance on neighboring towns and municipalities or, in some cases, international aid. In countries with limited behavioral health resources, primary care personnel and others within the community (teachers, first responders, family members, etc) will need to be increasingly relied upon to support response and recovery interventions.

#### Early Interventions

Early interventions found to be effective in the treatment of mass trauma include promoting safety, enhancing calming, increasing self- and community-efficacy, encouraging social connectedness, and engendering a sense of hope or optimism. Collectively, these have been termed Psychological First Aid (PFA). PFA serves as a framework for interventions designed to support the well-being of individuals and communities in the aftermath of traumatic events. It is a “do no harm” approach intended to be easy to use, simple to implement, and accessible to community members, rather than requiring delivery by healthcare providers. These principles have strong expert consensus as the most effective interventions following psychological trauma (69).

Promoting safety is established by removing individuals from immediately traumatic experiences and protecting them from secondary traumatization. Calming involves reducing arousal symptoms through relaxation techniques as well as providing information about assessment and management of the traumatic experience. Self- and community-efficacy enables the individual and communities to identify ways they can mitigate reactions and take a proactive role in their recovery from trauma. Connectedness reinforces existing social support networks and helps the individual build additional systems of support (friends, neighbors, relatives) where appropriate. Hope and optimism remind the individual that reactions and symptoms are a normal response which is expected to diminish over time and, when needed, additional resources will be made available. Online resources that are readily available, easy-to-read, and highly actionable can aid leaders, managers, and co-workers in responding to trauma in the workplace (70). Mobile resources and web-based training in PFA can help healthcare personnel enhance their skills in trauma response (71, 72).

While much is known about the role these five essential elements play in mental health outcomes after disaster, much less is known about effective methods of delivering the interventions, which interventions are most helpful for which people, and the timing with which interventions that promote any given essential element should be delivered (ie, is most important to first promote safety, calming, connectedness, etc)?. Dieltjens and colleagues conducted a systematic review of PFA-based interventions and concluded there was insufficient evidence to develop clinical practice guidelines based on PFA (73). Given the complexity of designing and implementing rigorous studies of PFA programs in real-world disaster settings and the variation in different PFA-based programs, a lack of compelling evidence for any specific intervention program is not unexpected. Future research should help clarify which interventions are most helpful for any given individual and community and at what point in time these are best delivered. A patient-centered, precision medicine approach might use crowd-sourcing, mobile device input and tracking, and big data analysis techniques to develop more tailored approaches for the delivery of early interventions.

Psychological debriefings, a central element of “Critical Incident Stress Management”, are still utilized in some settings with the goal of reducing adverse outcomes. However, most research indicates that debriefings following exposure to trauma do not reduce adverse psychological or behavioral effects and may increase risk for certain individuals (74). These debriefings should be avoided, particularly for unrelated and differently exposed groups, in favor of interventions based on the principles of PFA.

The additional treatment modalities listed subsequently in this paper all serve the purpose of addressing one or more of the five essential elements of PFA. Those working with communities impacted by disaster should consider the degree to which any additional interventions being considered effectively fit within the framework of PFA principles.

#### Behavioral Techniques

Well-established behavioral interventions that reduce physiological arousal include diaphragmatic breathing, progressive muscle relaxation, and guided visual imagery. These interventions facilitate the essential element of calming and may directly improve distress responses such as anxiety and insomnia (75, 76). They are easy to learn and simple to perform. These interventions can be taught by lay people or disaster response personnel with limited or no healthcare training, either face-to-face and/or through telephonic or online resources. Other benefits include being easily accessible, having little or no side effects, and increasing self-efficacy. Distressed individuals can also utilize these resources without risking stigmatization through seeking formal care. These interventions can be used in conjunction with other psychotherapy, pharmacotherapy, or complementary and alternative treatments.

#### Psychotherapy

Trauma-focused psychotherapies, such as Cognitive Processing Therapy and Prolonged Exposure Therapy, have the strongest evidence of benefit in the treatment of trauma and stressor-related PTSD. Trauma-focused psychotherapies incorporate imaginal exposure to the traumatic event in conjunction with an examination of cognitions the person may have about aspects of the event and their meaning. Negative thoughts such as “It's all my fault”, “If only I hadn't said something then this wouldn't have happened”, and other distorted cognitions are examined in collaboration between the individual and their treating healthcare provider. Subsequently, alternative and more balanced thoughts are considered and eventually used to replace the distressing negative thoughts. Trauma-focused psychotherapies also incorporate real-world behavioral interventions to assist individuals in overcoming avoidant behaviors and preserving social and occupational functioning. While trauma-focused therapy has been found highly successful in ultimately alleviating symptoms, an ongoing challenge for the healthcare field is maintaining patient engagement in this intervention. Barriers include frequent initial worsening of symptoms, time commitment, and patients' who are responding to the intervention maintaining motivation for treatment to full remission. The use of collaborative care models offers promise for enhanced patient engagement in primary care settings (77). Future research should seek to better understand efficacy of this model of care delivery and explore additional mechanisms for optimizing patient adherence to evidence-based psychotherapies.

#### Pharmacotherapy

Pharmacotherapy immediately following a disaster should generally be time-limited and symptom focused. Insomnia is a nearly universal symptom following a traumatic event. Because regulating sleep is critical to reducing arousal symptoms (and promoting the “calming” element of PFA), short-term sedative-hypnotic medication may be used to relieve insomnia. Eszopiclone (Lunesta) and Zolpidem (Ambien), both of which enhance GABA activity, are commonly prescribed for initiation insomnia. Prazosin (Minipress), an alpha-adrenergic blocker has some demonstrated efficacy in treating insomnia associated with posttraumatic symptoms as well as reducing the frequency and severity of associated nightmares and may be used at doses up to 15 mg nightly (78). Individuals with co-morbid depression may benefit from the sedating histamine properties of Trazodone (Oleptro), originally developed as a serotonin reuptake inhibitor for the treatment of depression. Medication for sleep should be provided in conjunction with additional interventions to promote sleep hygiene and address the range of PFA principles. As with all interventions, medication should be tailored to the individual's preference.

For those who develop psychiatric disorders (such as PTSD, depression, or anxiety) following a disaster, evidence-based pharmacotherapy includes SSRIs and SNRIs as first-line therapy. Mirtazipine (Remeron) also shows evidence of efficacy in treatment of PTSD. Prazosin has some support for treatment of PTSD-associated nightmares. Benzodiazepines (Valium, Klonopin, Xanax, and others) have primarily negative evidence and are generally contraindicated (79).

#### Complementary and Alternative

Complementary and alternative approaches to the treatment of traumatic stress have an increasing body of research supporting their efficacy and preliminary studies, as well as anecdotal evidence of benefit, are promising (80). Additional research on these interventions has focused on reducing anxiety and physiological arousal, both of which are helpful after highly distressing events. These interventions are increasingly sought out by patients as alternatives to traditional biological interventions. Individuals commonly report a desire for options that enhance self-efficacy and reduce the incidence of side effects as rationale for using these modalities. Mindfulness practices have the most robust research base to support their efficacy. Mindfulness is the practice of purposefully focusing on what is going on the present moment without passing judgment. It requires one to attend to thoughts, feelings, or sensations without resisting or trying to change them. This practice has generated increasing attention as an intervention to reduce stress and anxiety and improve functioning (81). Yoga has demonstrated early evidence in reducing adverse effects of trauma and related symptoms (82). Meditation and acupuncture are additional alternatives that should be considered. Animal-assisted therapy has become increasingly popular in the management of a range of psychological and behavioral symptoms, including those associated with traumatic stress as well as anxiety and other disorders. Animals may assist individuals who would otherwise be reluctant to engage in social activities following a traumatic event; thus, enhancing the critical treatment intervention of social connectedness. Individual's preference is an important determinant in considering whether to offer interventions currently considered complementary and alternative.

#### Barriers to Care

Limited availability of mental health care is common, particularly in low- and middle-income countries (LMICs). The World Health Organization found in 2017 that low income countries had 0.5 Psychiatrists per 100,000 members of the populations, compared to high income countries in which the ratio was 12.7 per 100,000 (83). Similar limited resources exist for other mental health professionals, such as Psychologists, Social Workers, and Psychiatric Nurse Practitioners. In the absence of international support, many citizens in LMICs will have no access to mental health care, making the knowledge of disaster mental health principles even more critical for primary care and emergency providers where many citizens will receive healthcare. Some will perceive distress reactions and psychiatric disorders as spiritual or religious manifestations and seek faith healers to address them; an opportunity for collaboration with community leaders to enhance access to healthcare interventions.

In spite of increased awareness and understanding of mental health, stigma continues to serve as a barrier to help-seeking. Stigma may be internal phenomenon in which an individual's negative perception of help-seeking leads them to avoid seeking care. Organizations and communities can also foster a culture which stigmatizes the use of help-seeking resources. Subtle or overt messages from friends, family, or co-workers may signal judgment or mistrust of those who use mental health or other help-seeking resources. Additional barriers include inadequate knowledge about available resources as well as lack of confidence in the efficacy of these resources.

## Special Topics

### Risk and Crisis Communication

Communication is a critical public health intervention tool in anticipation of and response to disasters. Risk and crisis communication shape public perceptions and impact community behaviors (84, 85). Communication during times of crisis helps build public trust, enhances participation in critical health-promoting behaviors (ie, evacuation, shelter in place, social distancing), reduces distress, and fosters cohesion within communities. Leaders at various levels should use established principles and techniques of risk and crisis communication to develop initial and ongoing public health messaging (86). Basic principles include telling the truth, saying what is known and unknown, committing to answering questions and following up, avoiding false reassurances, and using language that can be understood by the intended audience. Highly technical language, vague and evasive responses, and unprepared communicators lead to public confusion and increase mistrust. Important questions about public safety and what is being done to manage the impact of disaster for all those affected are predictable and communicators should be prepared to address these and provide ongoing updates at established intervals or more frequently if the situation dictates. When the public trusts those delivering messages, understands information provided, and believes that disaster response resources are being provided in an equitable manner, compliance with recommended public health behaviors increases.

Disasters that are protracted or which evolve over time (ie, Ebola outbreak in 2014, Washington DC sniper shootings in 2002) create unique communication requirements because perception of risk alters as the public's understanding of the event evolves. Perception of risk is an important determinant of community behavior. In a study of Arizona residents during the 2009 H1N1 outbreak, higher levels of perceived concern about H1N1 were associated with increased engagement in precautionary behaviors (87). During evolving ecological disasters, communication should be ongoing and routinely updated with the goal of optimizing community participation in public health behaviors that reduce exposure to harm. Though some messages will need to be delivered more spontaneously, leaders who prepare in advance and use established principles of risk and crisis communication for public health messaging are likely to most positively influence community behaviors.

Communication should also take into consideration those populations that may have difficulty accessing or understanding traditional information messaging. Those with cognitive impairment, institutionalized individuals, persons with different primary language from the majority community, as well as hearing and vision impairment are factors that should influence development and delivery of messages. Leaders and others crafting messages designed to influence public behavior and enhance disaster response and recovery should engage with community leaders and representatives to better understand unique factors that should be considered within any given population.

### Role of Leadership

While most research has been conducted in the aftermath of community violence, existing literature and experience of disaster managers demonstrates that leader behaviors have a significant impact on the recovery of communities and organizations following disasters (88). Fear of uncertainty may lead to avoidance of communication about the event. If a leader feels unprepared to support their community following a disaster, they should seek consultation from peers with prior experience as well as communications experts. Poor or absent leadership may lead community members to experience greater feelings of isolation, increase distress, and prolong impairment.

Leaders play an important role in reducing harm and mitigating the impact on individuals as well as the broader workplace community (89). Though some are expected to serve as leaders by virtue of title or position, informal leaders often emerge within communities. In 2017, Hurricane Harvey made landfall in Texas and devastated portions of the Gulf Coast, ultimately killing more than 100, displacing over 30,000 people, and causing $125 billion in damages. Jim McIngvale, known within the community as “Mattress Mack” used his furniture store to house displaced residence, as a location for delivery and pickup of critical supplies, and a focal point for rescue efforts (90). His leadership created a safe haven for displaced citizens, facilitated social connections for residence, and served as a beacon of hope. These informal leadership behaviors occur spontaneously and ideally augment the behavior of more formally established leaders throughout the community. There are a number of helpful interventions leaders can take following a community disaster (see **Table 8**).

**Table 8:** Interventions for leaders following a community disaster.

Speak directly with those involved about how they are doing
Include open-ended questions (“How are you doing?”, “What can I do to help?”)
Ensure individuals are aware of normal responses to trauma, when and where to get help
Be visible throughout the community by visiting people in-person whenever possible
Create time and space for community members to informally support one another
Openly and regularly communicate with the public about disaster response efforts
Publicly and privately discourage gossip, scapegoating, or other divisive behaviors
Model self-care by taking breaks, eating, hydrating, and getting sleep
Send clear messages the community supports and stands by affected individuals
Acknowledge inevitable grief and loss, while expressing optimism things will improve

Communication marked by active listening, empathy, support, and a desire to help reduces feelings of fear and isolation. In this way, leaders can provide the initial support to individuals impacted by disasters, a critical element in reducing distress and promoting recovery. Another key aspect of this communication is that leaders convey that distress reactions are acceptable, and that the community can and will support people through the difficult times.

Leaders must also pay attention to their own distress reactions and health risk behaviors following traumatic events. Poor sleep, over-dedication to the point of exhaustion, or withdrawal from their leadership role will have a negative effect on coping within the community. Leaders in these circumstances may feel isolated and, although they may be reluctant, should seek peer or expert consultation to assist in managing their own distress.

Grief is a near universal event following a traumatic event. There may be grief over loss of tangible items such as home, possessions, and cherished mementos. Community members may also experience feelings of loss regarding: 1) perception of their community as unsafe, 2) feeling unable to protect themselves, 3) diminished belief in their ability to be a productive member of the community. It is important for leaders to address grief and loss that arise following disasters, both for those who appear directly impacted as well as the broader community. Grief leadership is the process of recognizing and giving voice to what has been lost following traumatic events, providing a sense of hopefulness about recovery, a positive outlook on the future, and help community members begin the process of making meaning of the event (91). Effective grief leadership conveys and understands there may be adverse effects of a traumatic event and also that the individual is part of a community that desires to support them through the process of recovery.

## Conclusion

Ecological disasters can create profound disruption for communities that extends far beyond the geographic boundaries of the event. Psychological and behavioral responses create the most significant public health burden following a disaster. An understanding of community responses and the cultural and contextual factors that influence their development and evolution are critical for effective response and recovery efforts. Community response to extreme events show phases; an understanding of these optimizes timing and resourcing of recovery efforts. Interventions should be evidence-based, tailored to community needs, and serve to enhance the essential elements of safety, calming, self- and community-efficacy, social connectedness, and hope or optimism. Risk and crisis communication can shape community behaviors and influence perception of risk with trust and health-promoting behaviors being heavily influenced by thoughtful public health messaging. Effective leadership involves communication with community members, being present, honest, and trustworthy, modeling self-care, addressing community challenges such as grief and loss, and is essential for community recovery.

## Author Contributions

JM is the primary and corresponding author who developed the manuscript outline and used professional expertise to develop the initial content in draft form. RU oversaw the development of the manuscript outline and provided professional expertise in adding and modifying content for final submission.

## Disclaimer

The views expressed are those of the authors and do not necessarily reflect the views of the Department of Defense, the Uniformed Services University, the Department of Health and Human Services, or the United States Public Health Service.

## Conflict of Interest

The authors declare that the research was conducted in the absence of any commercial or financial relationships that could be construed as a potential conflict of interest.
